# Addendum: Clinical trial registration number for interventional health studies

**DOI:** 10.4069/kjwhn.2023.03.21

**Published:** 2023-03-31

This addendum was issued to note clinical trial registration numbers for the following publications.

In line with strengthening journal guidelines on adhering to the ICMJE guidelines on specifying clinical trial registration for interventional studies, the following manuscripts declare their clinical trial registration numbers. Prospective registration was not mandated by the funding agencies at start of the study and thus, retrospective registration was done by the author(s).

Koh M, Kim J, Yoo H, Kim S, Ahn S, Development and application of a couple-centered antenatal education program in Korea. Korean J Women Health Nurs. 2021;27(2):141-152. https://doi.org/10.4069/kjwhn.2021.06.20

The clinical trial registration number of above article is KCT0008065.

The clinical trial registration number has also been updated to the manuscript PDF and online access.

*Korean Journal of Women Health Nursing* strongly recommends prospective registration of interventional health studies.

